# A case of perforation of a pancreatic duct by a pancreatic stent during chemoradiotherapy for pancreatic head cancer: a case report

**DOI:** 10.1186/s40792-019-0571-3

**Published:** 2019-01-23

**Authors:** Takuya Mori, Go Ohira, Kenjiro Kimura, Sadaaki Yamazoe, Ryosuke Amano, Masaichi Ohira

**Affiliations:** Department of Surgical Oncology, Osaka City University Graduate School of Medicine, 1-4-3 Asahimachi, Abeno-ku, Osaka, 545-8585 Japan

**Keywords:** Pancreatic injury, Perforation of a pancreatic duct, Pancreatic stent, External drainage

## Abstract

**Background:**

Pancreatic injuries are rare, and no treatment plan has yet been established for grade III injuries. In many cases, pancreatic stent placement has resulted in saving patients. However, some cases of perforation of a pancreatic duct during the placement of a stent have been described, and there are also a few cases of delayed perforation by a pancreatic stent.

**Case presentation:**

A 62-year-old man had obstructive jaundice and pancreatitis due to locally advanced pancreatic head cancer. Both biliary and pancreatic stent were placed by endoscopy, after which chemoradiotherapy was performed. Four months later, he visited our hospital with severe abdominal pain. We performed enhanced CT and diagnosed the patient as having a perforation of a pancreatic duct by a pancreatic stent; therefore, we performed an emergency operation. Since we deemed pancreatectomy risky, we inserted pancreatic tubes into both sides of the perforated site and performed percutaneous transgastric drainage. The postoperative course was uneventful. We thereafter cut the tubes and switched to internal drainage.

**Conclusion:**

Many cases of pancreatic injuries have reported that pancreatic stent placement results in saving the patient, but there have been few cases in which a pancreatic stent causes perforation of a pancreatic duct. External drainage by pancreatic tubes is very effective in resolving perforation of a pancreatic duct.

## Background

Pancreatic injuries are rare, and no treatment plan has yet been established for grade III injuries (grade III on the American Association of Surgeons for Trauma [AAST] scale) [1]. In many cases, pancreatic stent placement has resulted in saving patients. However, some cases of perforation of a pancreatic duct during placement of a stent have been described, and there are also a few cases of delayed perforation by a pancreatic stent.

We herein report a case of perforation of a pancreatic duct after pancreatic stent placement, along with some literature review.

## Case presentation

A 62-year-old man had obstructive jaundice and pancreatitis due to locally advanced pancreatic head cancer (T3, N1, M0, stage IIB, TNM classification on UICC). Endoscopic placement of a biliary and pancreatic stent (plastic stent, 5Fr. 9 cm) was performed, after which chemoradiotherapy (20 Gy, gemcitabine 1353 mg/body+S-1 120 mg/body) had been performed for 5 weeks. Four months later, he suddenly developed severe abdominal pain with symptoms of peritoneal irritation and presented to our hospital. His blood pressure was 91/67 mmHg, pulse rate 113/min, and temperature 37.0 °C. His abdomen was hard with some tenderness.

**Fig. 1:**
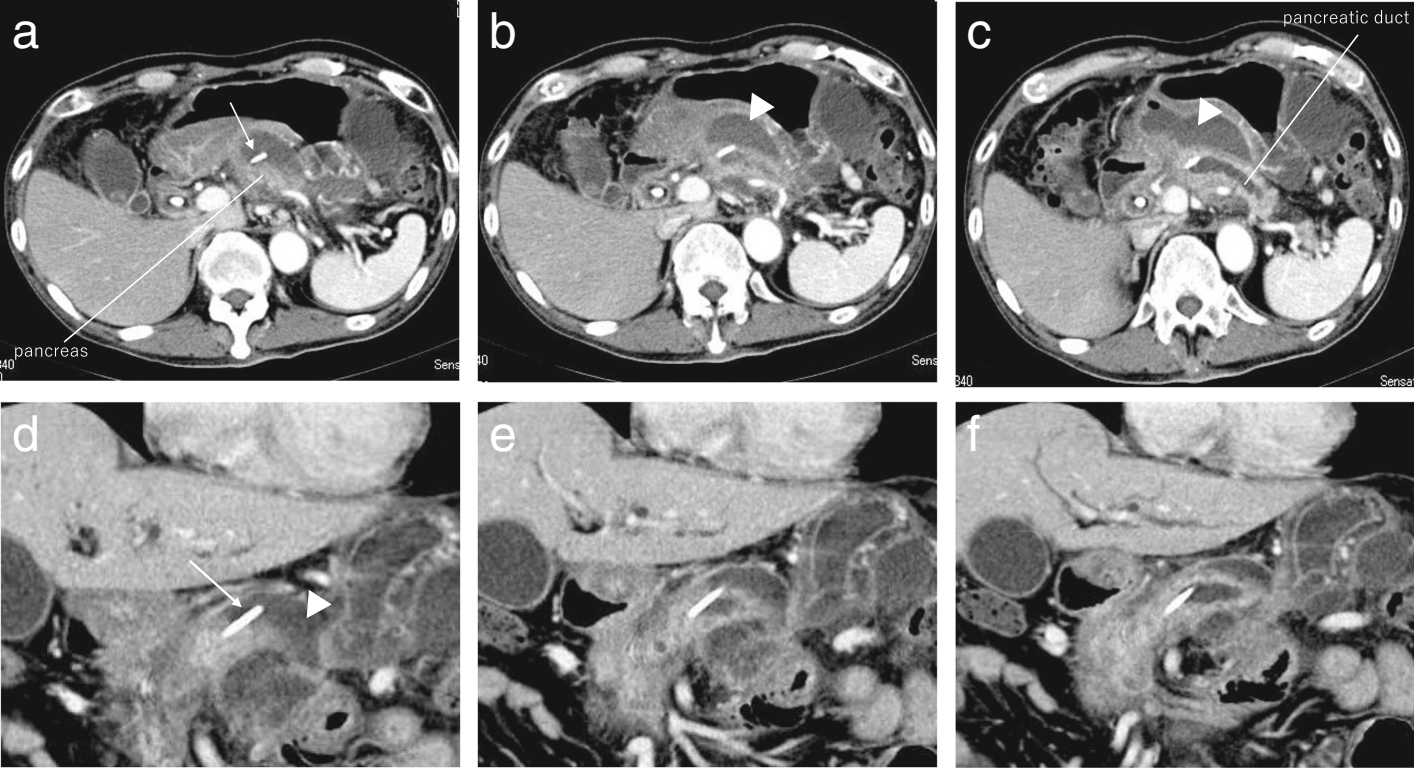
Enhanced computed tomography of the abdomen. A pancreatic stent protruded from the pancreatic body (arrow), and there was fluid collection around the greater omentum (arrowhead). **a**–**c** Axial view. **d**–**f** Coronal view

Laboratory data showed elevation of leukocytes (10,100/μl; reference values 4300 to 8000/μl) and C-reactive protein (13.92 mg/dl; reference values 0 to 0.40 mg/dl). Computed tomography (CT) revealed the tip of a pancreatic stent protruding from the pancreatic body, and there was fluid collection around the pancreas, omental bursa, and Douglas cavum (Fig. 1). A diagnosis of panperitonitis due to perforation of the pancreatic duct was confirmed, and emergency operation was performed. The onset time was unclear, but he had experience slight epigastric pain 1 week before visiting, and the possibility that this event had occurred approximately 1 week prior to presentation was thus considered.

**Fig. 2:**
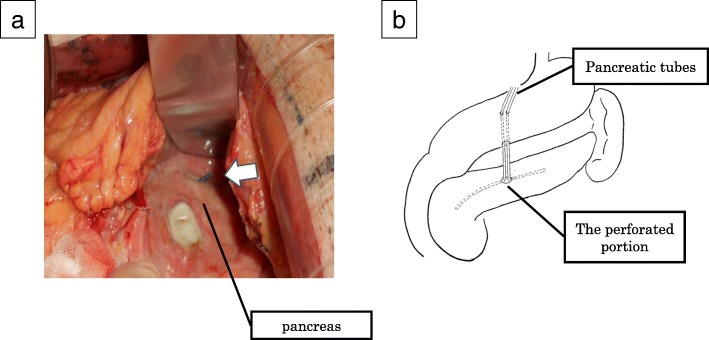
Operative findings. **a** A pancreatic stent protruded from the pancreatic body (arrow). **b** Pancreatic tubes were inserted into both sides of the perforated site, and external drainage was performed through the stomach. We performed percutaneous transgastric drainage

There was a large amount of cloudy ascites, and the tip of the pancreatic stent protruded from the pancreatic body (Fig. 2a). We deemed pancreatectomy and anastomosis to be risky with regard to postoperative complications. Therefore, we inserted pancreatic tubes into both sides of the perforated site, sutured between the posterior wall of the stomach and pancreas, and thereafter performed percutaneous transgastric drainage (Fig. 2b). The operation time was 173 min. The postoperative course was uneventful, and we changed to internal drainage by cutting the tubes in the stomach. The patient was discharged on postoperative day 87 and has undergone chemotherapy.

## Discussion

Perforation of a pancreatic duct during pancreatic stent placement is uncommon. Rashdan reported that only 3 of 2283 patients (0.1%) developed perforation of a pancreatic duct within 1–3 days after endoscopic retrograde cholangiopancreatography (ERCP) [2]. However, there have been no cases of perforation of a pancreatic duct several months after pancreatic duct stenting. In the present case, we speculate that stent attachment to the wall of a pancreatic duct was one of the causes of the perforation. In addition, chemoradiotherapy for pancreatic cancer may have made the pancreatic parenchyma brittle.

Pancreatic injuries are not frequent and are often associated with intra-abdominal injuries [3], occurring in only 3 to 12% of all patients with severe abdominal injuries [4]. Pancreatic injuries carry high mortality and morbidity rates, especially grade III (AAST scale) or worse injuries [5], so an early diagnosis and adequate therapy for pancreatic injuries is important.

CT is the most useful modality for diagnosing pancreatic injuries and can detect parenchymal lesions, but ductal disruption is commonly missed [6]. Endoscopic retrograde pancreatography (ERP) is one of the most useful methods for demonstrating the main pancreatic duct (MPD). Many recent reports have stated that pancreatic stent placement after a diagnosis by ERP prevents surgical treatment [5]. ERP has a high rate of complications (5–15%), such as pancreatitis, cholangitis, and duodenal perforation, but the importance of ERP is nevertheless increasing [7]. In this case, a broad abscess was found to have formed and the patient’s condition was poor; therefore, we thought that a surgical approach would be better than ERP in order to ensure the patient’s survival.

Surgical treatments for pancreas lesions vary, and the site, type, and surgeon’s experience are important for determining the most appropriate strategy. For grade III injuries, surgical treatment, such as pancreatic resection, can be selected. In pancreatic head injuries, pancreatoduodenectomy (PD) can be considered [8]. However, as PD may be accompanied by complications after surgery, we should select PD after consideration of the patient’s general condition and the surgical skill of the operator. In pancreatic body and tail injuries, distal pancreatectomy (DP) and splenectomy are the standard choice [9]. Letton-Wilson’s procedure, which consists of the closure of the proximal pancreatic segment and pancreato-jejunal anastomosis for the distal pancreatic segment, is one method that preserves the pancreas. However, this procedure is not generally recommended, as it can easily cause anastomotic leakage and result in the formation of pancreatic cysts [10]. The Bracy procedure, which is pancreato-gastric anastomosis, is said to cause less anastomotic leakage than other procedures because the pancreatic juice is not activated in the stomach [11]. Suturing the pancreatic duct is the ideal surgical procedure, but it is very difficult, and there is a high risk of pancreatic fistula.

The concept of damage control surgery (DCS) has also been accepted. For example, the external drainage of the MPD and towel packing against the bleeding point can be performed. Patton et al. [12] reported that simplified external drainage was successful with low morbidity and mortality in cases of severe pancreatic injury.

In the present case, the pancreatic body was damaged, and while DP was indicated, we judged pancreatic resection to be unsuitable. First, we planned to perform PD for pancreatic head cancer in the future, so consequently, we wanted to preserve the distal pancreas. Second, because the patient was receiving chemoradiotherapy for cancer, we suspected that the pancreatic parenchyma and surrounding tissue might be fragile. Finally, panperitonitis due to pancreatic fistula occurred, and this patient’s general condition was poor. For these reasons, we selected the external drainage of a pancreatic duct. In addition, the distance between the posterior wall of the stomach and the perforated site of the pancreas was very small, and thus, it was easy to pass the tubes into the stomach, and we thought the perforated site was covered with the stomach. Therefore, we performed transgastric drainage. Regarding why the patient has had such a good postoperative course, we think that the size of the pancreatic tube matched that of the pancreatic duct well and the pancreatic tube passing through the stomach resulted in a setup similar to pancreato-gastric anastomosis. Although transgastric external drainage is not always useful in general pancreatic injuries, we think that this procedure should be considered as a choice in systemically unstable patients.

## Conclusion

We experienced a case in which a pancreatic stent caused perforation of a pancreatic duct, and we performed transgastric external drainage. Pancreatic injuries are rare, and it is necessary to determine the surgical procedure with careful consideration of the patient’s condition and the operator’s skill. External drainage is a useful procedure in unstable patients. We should bear in mind that pancreatic stents can cause perforation even after a long time has passed following stent placement. We should also consider the site of stent placement and replace stents as needed.

## Abbreviations

AAST: American Association of Surgeons for Trauma
CT: Computed tomography
DCS: Damage control surgery
DP: Distal pancreatectomy
ERCP: Endoscopic retrograde cholangiopancreatography
ERP: Endoscopic retrograde pancreatography
MPD: Main pancreatic duct
MRCP: Magnetic retrograde cholangiopancreatography
PD: Pancreatoduodenectomy
